# The genus *Aphidura* (Hemiptera, Aphididae) in the collection of the *Muséum national d’Histoire naturelle* of Paris, with six new species

**DOI:** 10.3897/zookeys.318.5693

**Published:** 2013-07-23

**Authors:** Juan-Manuel Nieto Nafría, Milagros-Pilar Mier Durante, Georges Remaudière

**Affiliations:** 1 Departamento de Biodiversidad y Gestión Ambiental, Universidad de León, 24071 León, Spain; 2 Département de Systématique et Évolution, Muséum national d’Histoire naturelle, 45 rue Buffon, 75005 Paris, France

**Keywords:** New taxa, descriptions, synonymies, key of species, host plants, distributions

## Abstract

Specimens were studied of 65 samples of the genus *Aphidura* (Aphididae, Aphidinae, Macrosiphini) from the collection of the Muséum national d’Histoire naturelle (Paris). The possible synonymies of three pairs of species are discussed. New aphid host plant relationships are reported for *Aphidura bozhkoae*, *Aphidura delmasi*, *Aphidura ornata*, *Aphidura pannonica* and *Aphidura picta*; this last species is recorded for first time from Afghanistan. The record of *Aphidura pujoli* from Pakistan is refuted. The fundatrices, oviparous females and males of *Aphidura delmasi* are described. Six new species are established: *Aphidura gallica* **sp. n.** and *Aphidura amphorosiphon* **sp. n.** from specimens caught on species of *Silene* (Caryophyllaceae) from France and Iran, respectively, *Aphidura pakistanensis* **sp. n.**, *Aphidura graeca* **sp. n.** and *Aphidura urmiensis* **sp. n.** from specimens caught on species of *Dianthus*, *Gypsophila* and *Spergula* (Caryophyllaceae) from Pakistan, Greece and Iran, respectively, and *Aphidura iranensis* **sp. n.** from specimens caught on *Prunus* sp. from Iran. Modifications are made to the keys by Blackman and Eastop to aphids living on *Dianthus*, *Gypsophyla*, *Silene*, *Spergula* and *Prinsepia* and *Prunus* (Rosaceae). An identification key to apterous viviparous females of species of *Aphidura* is also provided.

## Introduction

In the early 1980s G. Remaudière and D. Hille Ris Lambers studied some samples of *Aphidura* (Hemiptera, Aphididae, Aphidinae, Macrosiphini) belonging to the Remaudière collection, which was at that time at the *Institut Pasteur* in Paris, but later moved to the *Muséum national d’Histoire naturelle*. They made preliminary works to describe several new species of this genus, but the work was interrupted and a manuscript draft was never prepared due to the illness and death in April 1984 of Hille Ris Lambers. Some of the slides studied by Hille Ris Lambers were sent with the rest of his collection to the British Museum (Natural History), currently the Natural History Museum, in London. Some years later Remaudière did establish one new species in this genus (Remaudière 1989) but this was from specimens that had not been part of his studies with Hille Ris Lambers.

All those samples, together with the rest of the specimens of *Aphidura* of the above-mentioned collection, have now been studied, and the results are presented in this paper.

The genus *Aphidura* was morphologically well defined by Hille Ris Lambers (1956), and the type species, *Aphidura ornata* Hille Ris Lambers, is also well defined, so no doubt exists about the correct taxonomic position of the species included within it. Apterous viviparous aphids can be identified as *Aphidura*, in addition to the characteristics of the tribe Macrosiphini, by the presence of a pair of mesosternal mammariform processes (in Hille Ris Lambers’s words: «*Distinct, conspicuously pigmented or pale, rough mammiform processi present on anterior part of mesosternum in apterae and larvae*»), together with a triangular or tongue-shaped cauda and reniform spiracular apertures centred in the respective sclerites.

Similar mesosternal processes are also present in several species of *Brachycaudus* van der Goot, 1913, mainly belonging to subgenus *Acaudus* van der Goot, 1913, and in the sole species of *Zinia*, *Zinia veronicae* Shaposhnikov, 1950. These species of *Brachycaudus* have a helmet-shaped cauda and wide, rounded spiracular apertures, and *Zinia veronicae* has a rounded cauda, reniform spiracular apertures that are placed in the posterior half of the spiracular sclerites and, in addition the dorsal cuticle is densely spinulose (Hille Ris Lambers 1956, Shaposhnikov 1950, Andreev 2004).

*Aphidura* currently includes 16 to 18 species and 1 subspecies (Blackman and Eastop 2006, Kadyrbekov 2013): *Aphidura acanthophylli* Remaudière, 1989, *Aphidura alatavica* Kadyrbekov, 2013, *Aphidura bozhkoae* (Narzikulov, 1958) (type species of *Cerasomyzus* Narzikulov, 1958, which was established as subgenus of *Myzus* Passerini), *Aphidura delmasi* Remaudière and Leclant, 1965, *Aphidura gypsophilae* Mamontova-Solukha, 1963, *Aphidura massagetica* Kadyrbekov, 2013, *Aphidura melandrii* Kadyrbekov, 2013, *Aphidura mordvilkoi* Shaposhnikov, 1984 (with its possible synonym *Aphidura prinsepiae* Pashchenko, 1988), *Aphidura naimanica* Kadyrbekov, 2013, *Aphidura nomadica* Kadyrbekov, 2013, *Aphidura ornata* Hille Ris Lambers, 1956, *Aphidura ornatella* Narzikulov & Winkler, 1960 (with its possible synonym *Aphidura bharatia* David, Sekhon & Bindra, 1970), *Aphidura pannonica* Szelegiewicz, 1967 (with subspecies *Aphidura pannonica cretacea* Mamontova-Solukha, 1968), *Aphidura picta* Hille Ris Lambers, 1956 (with its possible synonym *Aphidura mingens* Pintera, 1970), *Aphidura pujoli* (Gómez-Menor Ortega, 1950) and *Aphidura togaica* Kadyrbekov, 2013.

Two species of *Aphidura* live on Rosaceae species and other species live on Caryophyllaceae species, mainly belonging to genus *Silene*. *Aphidura pujoli* is monoecious holocyclic on Caryophyllaceae; it is possible that the life cycle of other species of *Aphidura* is also monoecious holocyclic, though it is also possible that some species host-alternate between species of Rosaceae and Caryophyllaceae (Blackman and Eastop 2006, Holman 2009, Kadyrbekov 2013).

The genus exhibits a Mediterranean-Pontian-Turanian distribution with extensions to neighbouring areas and exceptionally – *Aphidura mordvilkoi* – to the Russian Far East. The current known distribution of each species is shown in the species identification key at the end of this paper, *Aphidura picta* being the species with the widest distribution (Blackman and Eastop 2006, Holman 2009, Nieto Nafría et al. 2012, Kadyrbekov 2013).

## Material and methods

*Aphidura* specimens of the aphid collection of the *Muséum national d’Histoire naturelle of Paris*, mounted in microscopic slides, belonging to 65 samples (Table 1) have been studied.

Aphids were identified, or their previous identifications were checked, by reference to the original descriptions (Hille Ris Lambers 1956, Gómez-Menor Ortega 1950, Narzikulov 1958, Narzikulov and Winkler 1960, Mamontova-Solukha 1963, Remaudière and Leclant 1965, Szelegiewicz 1967, Mamontova-Sholukha 1968, David et al. 1970, Pintera 1970, Shaposhnikov 1984, Pashchenko 1988, Remaudière 1989, Kadyrbekov 2013) and other informative works (Eastop and Blackman 2005, Blackman and Eastop 1994, 2006).

**Table 1.** Studied samples.

***Aphidura* species** Host plant	**Country**	**Locality**	**Date**	**Coll.**	**Sample**
***Aphidura acanthophylli***					
*Acanthophyllum* sp.	Iran	Sharh-e Babak [NW 50 km] (Kerman)	4-IX-1972	R.	i3749
***Aphidura amphorosiphon* sp. n.**					
*Dianthus* sp.	Iran	without locality	sans date	D.	i1440
*Silene* sp.	Iran	Kuh-e Dinar (Kohgiluyed and Boyer-Ahmad)	14-IX-1955	R.	i1118a
Caryophyllaceae	Iran	Chalus [N 40 km Amol road] (Mazenderan)	3-V-1963	R.	i2417
***Aphidura bozhkoae***					
*Prunus spinosa*	Iran	Karadj (Alborz)	8-V-1955	R.	i196
*Prunus ?prostrata*	Iran	Bojnurd [E 10 km] (North Khorasan)	21-V-1966	R.	i2961
*Prunus ?prostrata*	Iran	Kuh-e Choret [90 km. Bojnurd] (North Khorasan)	25-V-1966	R.	i3028
*Prunus* sp.	Iran	?	?	?	i4347
	Iran	Shiraz (Fars)	?-V-1974	C.	i4092
***Aphidura delmasi***					
*Silene italica*	France	Gémenos (Bouches-du-Rhône)	13-VI-1967	R.	6455
*Silene italica*	France	Lantosque (Alpes-Maritimes)	24-X-1968	R.	7591
*Silene italica*	France	Saint-Guilhem-le-Désert (Hérault)	17-IV-1966	R.	5751
*Silene italica*	France	Saint-Guilhem-le-Désert (Hérault)	21-VII-1966	L.	5752
*Silene italica*	France	Saint-Guilhem-le-Désert (Hérault)	30-IX-1966	L.	5753
*Silene italica*	France	Pont du Gard (Gard)	19-III-1969	R.&L.	7728
*Silene italica*	France	Utelle (Alpes-Maritimes)	11-V-1969	R.&L.	7876
*Silene italia*	France	Utelle (Alpes-Maritimes)	13-VI-1988	R.	15798
*Silene ?viscosa*	France	Finistret (Pyrénées Orientales)	9-VI-1983	R.	14459
*Silene* sp.	Greece	Lagadie [East] (Akadia)	3-VII-1964	R.	03087
*Silene* sp.	France	Lantosque (Alpes-Maritimes)	28-II-1970	R.	9258
*Silene* sp.	France	La-Garde-Freinet (Var)	26-III-1970	R.	9357
*Silene* sp.	France	Saint-Jean la-Rivière (Alpes-Maritimes)	16-IX-1969	R.	8690
vagrant	France	Utelle (Alpes-Maritimes)	7-XI-1989	R.	16079 b
***Aphidura gallica* sp. n.**					
*Silene gallica*	France	Banyuls-sur-Mer (Pyrénées-Orientales)	11-VII-1957	R.	4241
*Silene paradoxa*	France	Defilé de l’Inzecca (Haute-Corse)	4-VI-1979	L.	17925
***Aphidura graeca* sp. n.**					
*Gypsophila* sp.	Greece	Veria [to Kastania] (Imanthia)	18-VI-1964	R.	03026
***Aphidura gypsophilae***					
*Gypsophila paniculata*	Slovakia	Chotín (Nitra)	25-VI-1984	H.	015379
***Aphidura iranensis* sp. n.**					
*Prunus* sp.	Iran	Khoy [N 30 km] (West Azerbaijan)	7-VIII-1955	R.	i982
***Aphidura mordvilkoi***					
*Princepia sinensis*	Russia	? (Prymorsky Krai)	20-VI-1967	Sh.	016559
*Princepia sinensis*	Russia	? (Prymorsky Krai)	5-VI-1980	Pa.	014789
***Aphidura ornata***					
*Silene inaperta*	France	Ste Catherine de Vars (Hautes-Alpes)	1-VII-1990	R.&M.V.	16454
*Silene italica*	France	Avène (Hérault)	1-V-1967	L.	18054
*Silene nutans*	Switzerland	Cassarate (Ticino)	25-V-1950	H.R.L.	02946
*Silene nutans*	Switzerland	Cassarate (Ticino)	25-V-1950	H.R.L.	016758
*Silene saxifraga*	France	La-Roche-de-Rame [S] (Hautes-Alpes)	22-VI-1969	R.&L.	8010
***Aphidura ornatella***					
*Silene* sp.	Pakistan	Matiltan (Khyber Pakhtunkhwa)	14-VIII-1991	N-E.	014109
trap	Pakistan	Kalam Khyber Pakhtunkhwa	?-?-1987	N-E.	
trap	Pakistan	Matiltan (Khyber Pakhtunkhwa)	23-VII-1987	N-E.	
trap	Pakistan	Matiltan (Khyber Pakhtunkhwa)	30-VII-1987	N-E.	
***Aphidura pakistanensis* sp. n.**					
*Dianthus* sp.-	Pakistan	Kalam (Khyber Pakhtunkhwa)	17-VIII-1981	N-E.	014072
***Aphidura pannonica***					
*Gypsophila paniculata*	Hungary	Ágasegyháza (Bács-Kiskun)	10-VI-1968	Sz.	014156
*Silene otites*	Hungary	Budapest [Sas-hegy] (Pest)	21-VI-1964	Sz.	014156
*Silene otites*	Slovakia	Chotín (Nitra)	25-VI-1984	H.	015380
*Silene otites*	Slovakia	Somotor (Košice)	27-VI-1962	Pi.	010615
***Aphidura picta***					
*Dianthus barbatus*	Pakistan	Quetta (Baluchistan)	14-V-1991	N-E.	013878
*Dianthus crinutus*	Pakistan	Skardu (Gilgit–Baltistan)	2-VII-1991	N-E.	013965
*Dianthus ?barbatus*	Iran	Isfahan (Isfahan)	25-IV-1978	R.	i4222
*Dianthus* sp.	Afghanistan	Kabul (Kabul)	26-VI-1972		04565
*Dianthus* sp.	Iran	Karadj (Alborz)	?-XI-1948	D.	i81a
*Dianthus* sp. [cult.]	Turkey	Ankara (Ankara)	8-X-1950	T.	011930
*Silene conoida*	Iran	Laleeh zar (Kerman)	26-VI-1955	R.	i648
*Silene fruticosa*	Italy	Castelmola (Messina)	9-VI-1979	B.	012841
*Silene glauca*	Spain	Callosa de Ensiarrá (Alicante)	29-V-985	G.F.	012841
*Silene italica*	France	Col Turini (Alpes-Maritimes)	15-X-1969	R.	8672
*Silene italica*	France	Lantosque (Alpes-Maritimes)	24-X-1968	R.	7592
*Silene* sp.	Iran	Karadj (Alborz)	19-V-1955	R.	i282c
***Aphidura pujoli***					
*Dianthus caryophyllus*	France	Defilé de l’Inzecca (Haute-Corse)	4-VI-1970	L.	18055
*Dianthus caryophyllus*	France	Defilé de l’Inzecca (Haute-Corse)	4-VI-1970	L.	18056
*Dianthus caryophyllus*	Italy	Ercolano [previously Resina] (Napoli)	17-VIII-1936	Ro.	02947
*Dianthus* sp.	Spain	Arenas de Cabrales (Asturias)	7-VI-1981	R.&N.N.	012841
*Dianthus* sp.	France	?	?	?	5670
trap	France	Montpellier (Hérault)	16-VII-1996	?	17749
trap	France	Valence (Charente)	1-VII-1996	?	17755
***Aphidura urmiensis* sp. n.**					
*Spergularia marina*	Iran	Charimboulaki, Lac Urmia (West Azerbaijan)	9-VIII-955	R.	i1004
*Spergularia marina*	Iran	Shahi island, Lac Urmia (East Azerbaijan)	5-VIII-1955	R.	i962

NOTES:<br/> In the “Locality” column, supplementary information and upper administrative unit (such as county, department, province, regional unit, etc.) are respectively given in square brackets and in parentheses.<br/> In the “Coll” column the names of collectors have been abbreviated as follows: B., Barbagallo (S.); C., Chodjaï (M.); D., Davatchi (A.); G.F., González Funes (M.P.); H.R.L, Hille Ris Lambers (D.); H., Holman (J.); L., Leclant (F.); M.V. Muñoz Viveros (A.L.); N.N., Nieto Nafría (J.M.); N-E, Naumann-Etienne (K.); Pa., Pashchenko (N.S.); P., Pintera (A.); R., Remaudière (G.); Ro., Roberti (D.) Sh., Shaposhnikov (G.C.); Sz., Szelegiewicz (H.); and T., Tuatay (N.).<br/> The numbers in the “Sample” column are the numbers of the Remaudière samples.

Morphological measurements were made according to Nieto Nafría and Mier Durante (1998). In the descriptions and keys, measurements are lengths except when indicated otherwise as width or diameter. A Leica DC digital 96 camera with IM 1000 version 1.10 software was used for the photomicrographs, which have been taken and mounted by L. M. Fernández Blanco.

In the modifications to the identification keys by Blackman and Eastop (1994, 2006) that are included in the discussion of each species, the terms that they use (for example ‘hair’ instead of ‘seta’ and ‘clavate’ instead of ‘swollen’) have been retained so that they can be easily understood and used by those accustomed to them.

## Results and discussion

### Generic characters

Apterous viviparous aphids can be identified as *Aphidura* by the presence of a pair of mesosternal mammariform processes, as mentioned above, and also by the following characters: (1) frons w-shaped with rugose or scabrous lateral tubercles not much higher than the broad median tubercle; (2) cephalic dorsum not ornamented or with spinules, which may be more-or-less scattered or in groups; (3) clypeus and mandibular and maxillar lamina more-or-less pigmented like cephalic dorsum and rostrum; (4) antennae not longer than body length; (5) secondary sensoria absent; (6) antennal segment I and II scabrous or rugose, segment III with scattered scales, and IV–VI more-or-less imbricated; (7) rostrum extending backward beyond middle coxae or reaching hind coxae; (8) ultimate rostral segment triangular with straight margins, usually darker than the previous segments; (9) legs with coxae and trochanters pale, femora entirely pale or with a darker distal part; tibiae pale in general with a distal portion smoky, exceptionally entirely pale, and tarsi brown; (10) first segment of tarsi with 2–4 setae; (11) abdominal spiracular apertures reniform, placed in the middle of small spiracular sclerites; (12) intersegmental sclerites well defined and usually pigmented, and embodied in the segmental sclerites if these are present; (13) thorax and abdomen often with a dorsal pattern of sclerotisation that is very variable between species, and can also vary within them (see below terminological usage); (14) siphunculi usually with a distinct preapical incision and flange, but variable in shape (see below for details and terminological usage); (15) cauda triangular to tongue-shaped; (16) spinules present, more-or-less conspicuously and densely, on mesosternal mammariform processes, postsiphuncular sclerites, spiracular sclerites 7, and abdominal terga 7 and 8; (17) antennal and dorsal setae short or very short, with blunt, frayed or (rarely) incrassated apex; (18) dorsal setae not placed on tubercles, except sometimes in *Aphidura acanthophylli*; (19) ventral setae longer than respective dorsal and pointed; and (20) setae on dorsal faces of femora and on proximal parts of tibiae with blunt or frayed apex, other setae on legs pointed.

The alate viviparous females have no mesosternal mammariform processes, and differ from apterae by having: (a) longer and more pigmented antennae, (b) round, double-rimmed secondary sensoria scattered along the ventral face of antennal segment III, and rarely on segment IV, (c) pigmentation of legs more extensive and darker; (d) dorsal abdomen often with more sclerotisation than in apterae, but again this varies greatly both between and within species; (e) spinules also present in the marginal sclerites.

**Figure 1.**
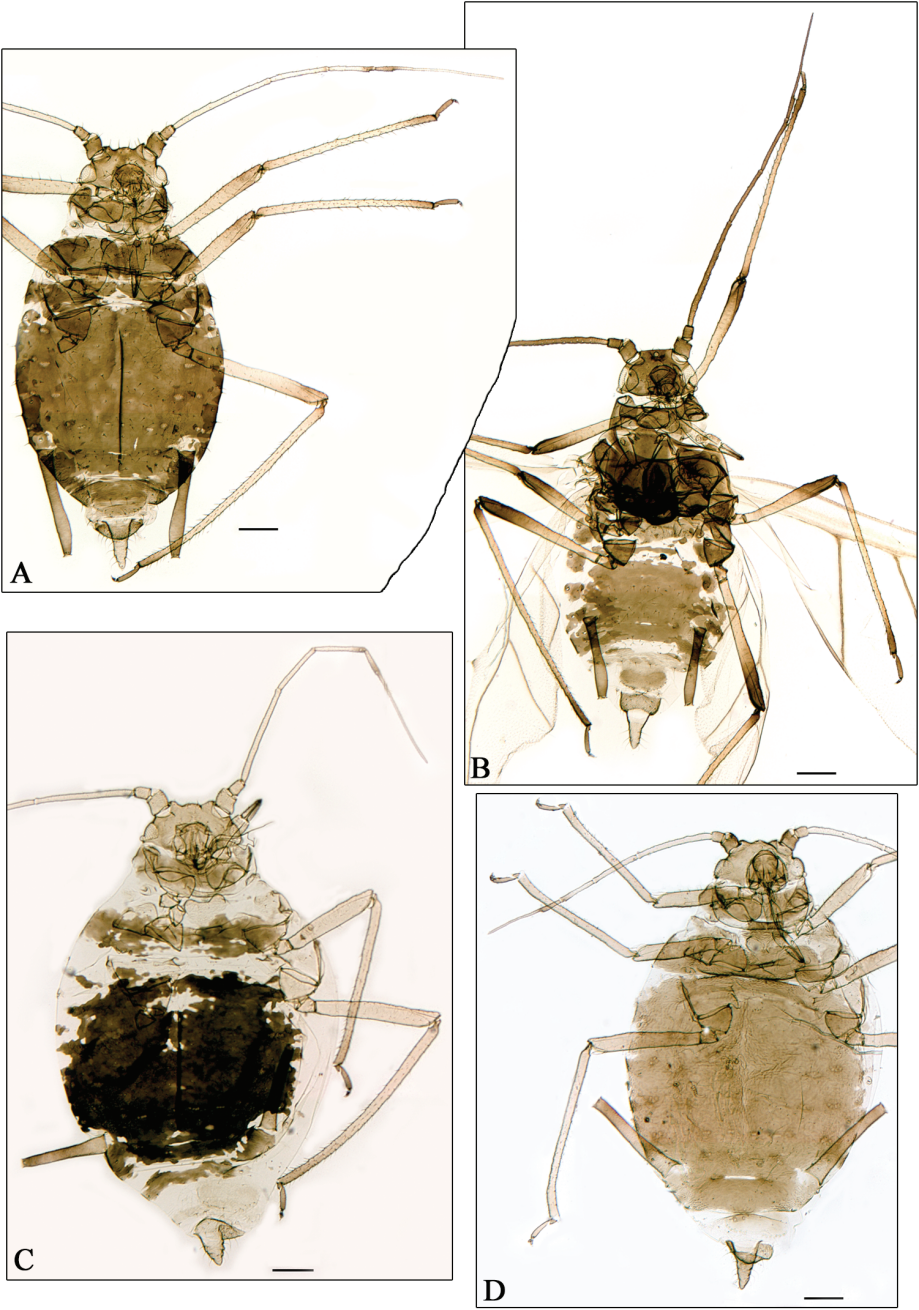
**A–B** *Aphidura ornatella* **C** *Aphidura picta* **D** *Aphidura mordvilkoi* **A, C–D** apterous viviparous female **B** alate viviparous female. Scale bars 0.2 mm.

**Figure 2.**
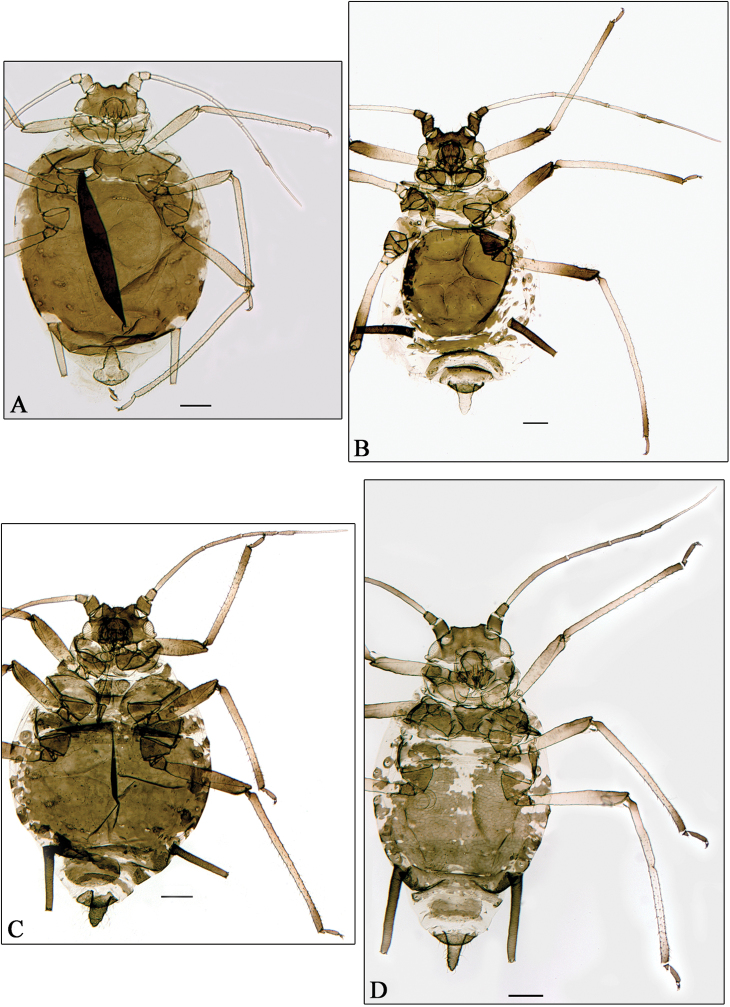
**A** *Aphidura bozkhoae*. **B** *Aphidura delmasi*. **C** *Aphidura ornata*. **D** *Aphidura pannonica*. **A–D** apterous viviparous female. Scale bars 0.2 mm.

Regarding the thoracic and abdominal dorsal sclerotisation, the term “spinopleural patch” is utilized here for a continuous sclerotisation of spinal and marginal areas of two or more segments (Figs 2B, D, 4A, 6A), and the term “discal plate” is utilized for the continuous and extensive sclerotisation of spinal, pleural and marginal areas of three or more segments (Figs 1A, C, 2A, C).

**Figure 3.**
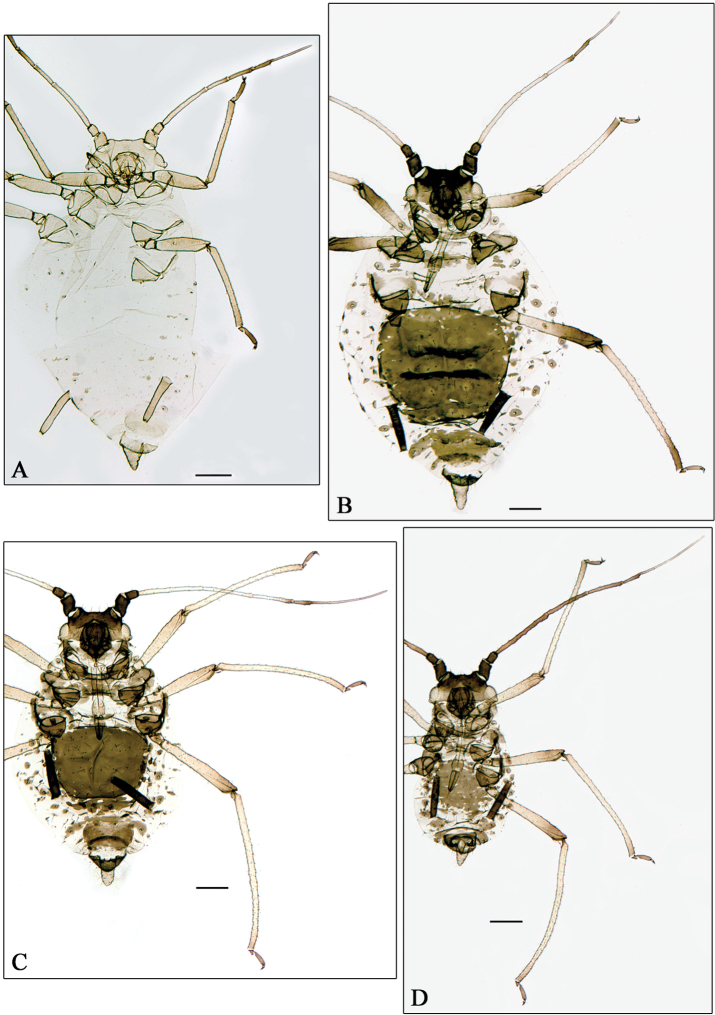
**A** *Aphidura pujoli*. **B–D** *Aphidura delmasi* **B** fundatrix **C** oviparous female **D** male. Scale bars 0.2 mm.

**Figure 4.**
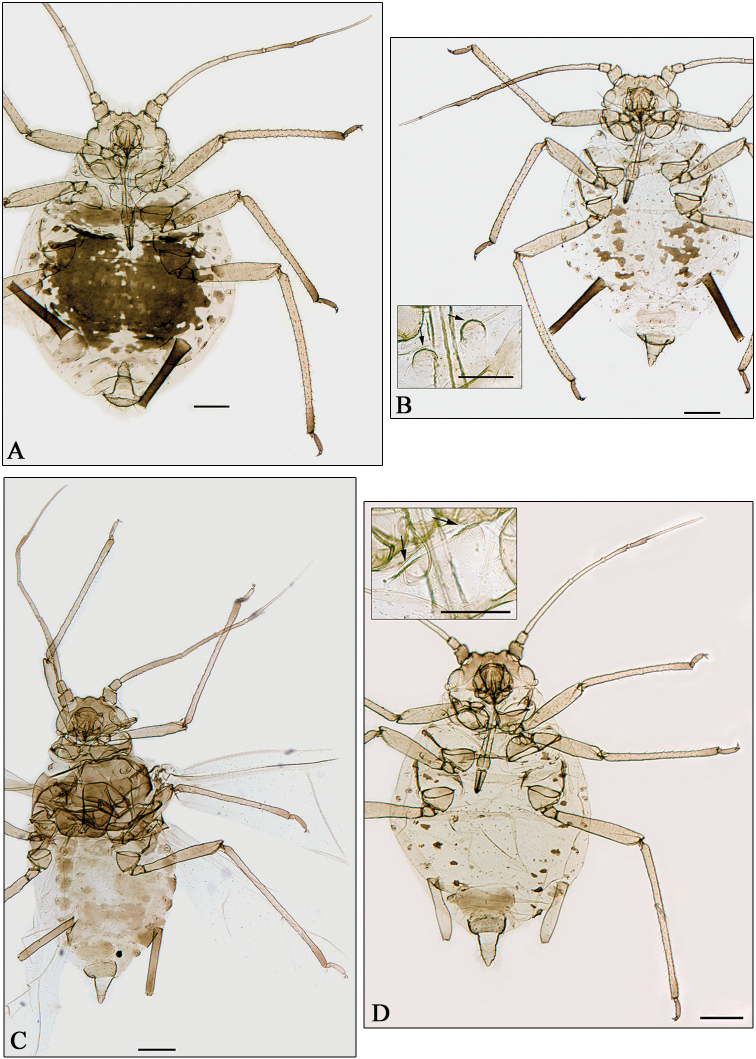
**A–C** *Aphidura gallica* sp. n. **D** *Aphidura amphorosiphon* sp. n. **A–B, D** apterous viviparous female **C** alate viviparous female**A** pigmented form**B** unpigmented form **B, D** boxes mesosternum with mammariform processes. General scale bars 0.2 mm, boxes scale bar 0.01 mm.

**Figure 5.**
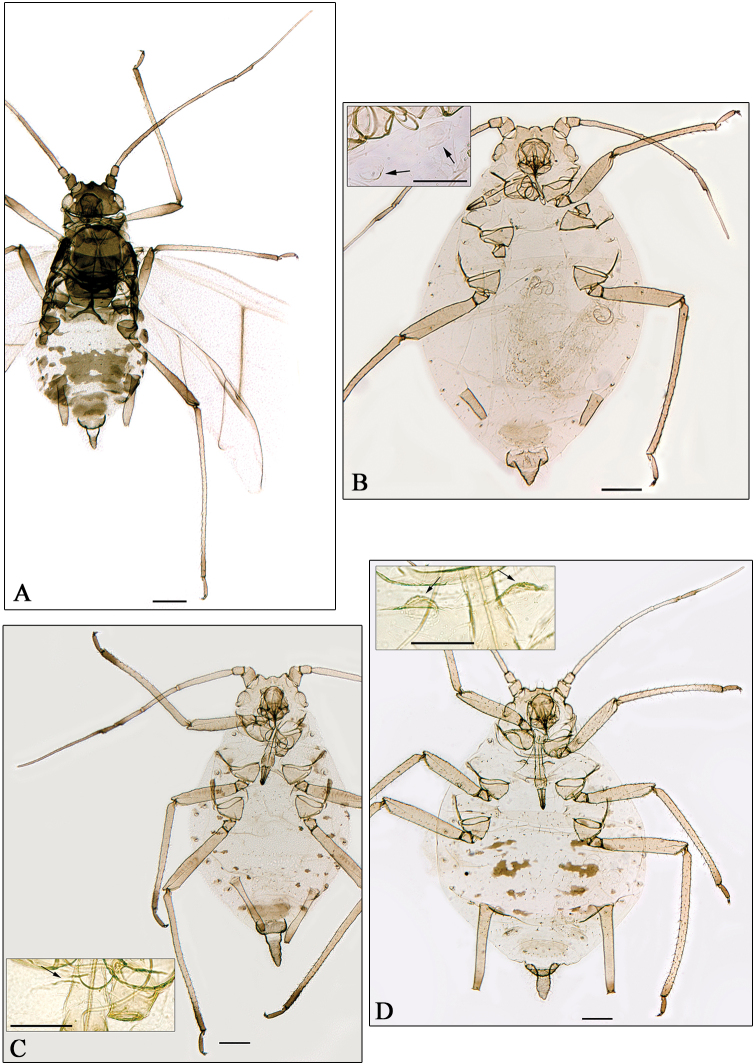
**A** *Aphidura amphorosiphon* sp. n. **B** *Aphidura pakistanensis* sp. n. **C** *Aphidura graeca* sp. n. **D** *Aphidura urmiensis* sp. n. **A** alatae viviparous female **B–D** apterous viviparous female **B–D** boxesmesosternum with mammariform processes. General scale bars 0.2 mm, boxes scale bar 0.01 mm.

**Figure 6.**
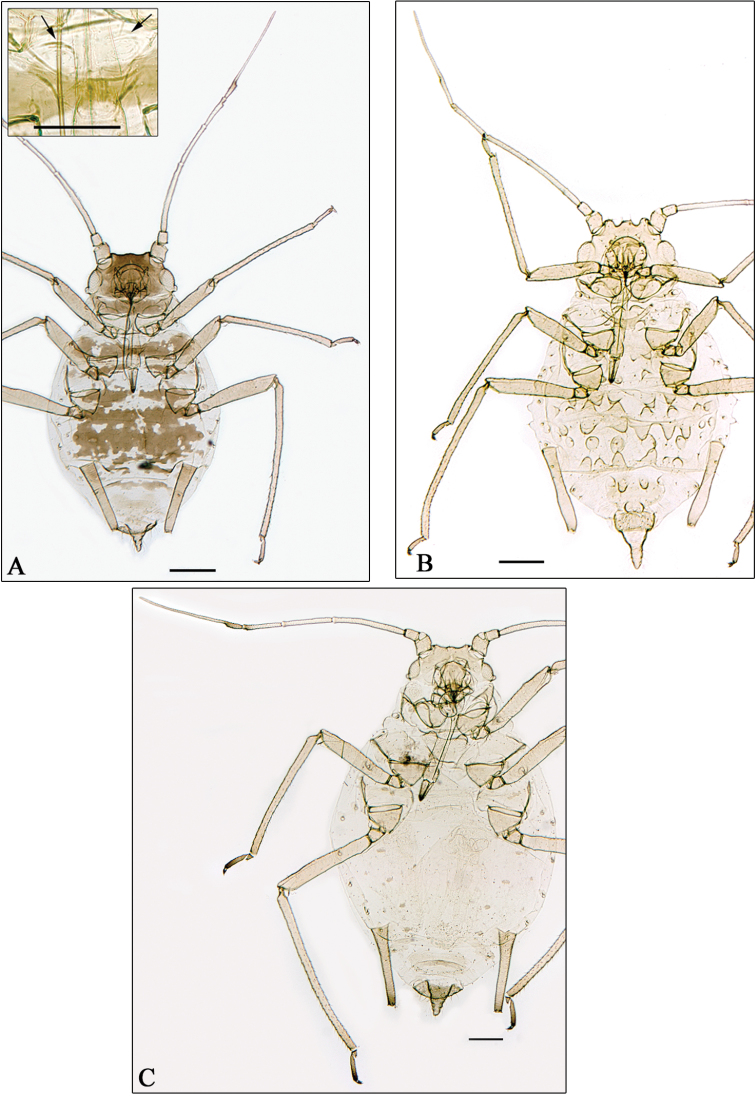
**A** *Aphidura iranensis* sp. n. **B** *Aphidura acanthophylli* **C** *Aphidura gypsophilae* **A–C** apterous viviparous female **A** box mesosternum with mammariform processes. General scale bars 0.2 mm, box scale bar 0.01 mm.

The siphunculi of *Aphidura* species are variable in shape: (a) cylindrical, subcylindrical (delicately tapering to the apex) or conspicuously tapering from base to apex, straight or curved outward (Figs 1D, 2A, 4B, 6A); (b) slightly swollen —«*cylindrical with very tapering apex, below their middle very little attenuated, so that they might be considered as very slightly clavate*» (Hille Ris Lambers 1956)—, having the maximal width of the distal half less than 1.2 times the minimal width of the stem (Figs 2D, 3A, 5B, 6A); (c) markedly swollen, with large base, cylindrical stem and a conspicuously swollen distal portion, the width of which is conspicuously greater (at least 1.2 times) than the minimal stem width (Figs 1A, 4D, 5C, 6B).

### Synonymies in *Aphidura*

Possible synonymies of three pairs of *Aphidura* species are discussed: *Aphidura bharatia* and *Aphidura ornatella*, *Aphidura mingens* and *Aphidura picta*, and finally *Aphidura prinsepiae* and *Aphidura mordvilkoi*.

Eastop and Blackman (2005) established that *Aphidura bharatia* is a synonym of the older name *Aphidura ornatella*. Both species are considered valid by Kadyrbekov (2013) because he found differences between aphids identified as *bharatia* and others identified as *ornatella*, although he does not refer to Eastop and Blackman’s paper. In our opinion these differences could be enough to keep the validity of both species, but they can also be an expression of intraspecific variability, which would present a cline North to South; Kadyrbekov himself shows differences between *ornatella* populations from Kazakhstan and Tajikistan.

Characters of studied apterous and alate vivipara (Figs 1A, B; Table 2 for six alatae from Pakistan; only one alate of this species was previously known [Kadyrbekov 2013]), overlap characters mentioned by Kadyrbekov for southern (from India and Pakistan) and northern (from Kazakhstan and Tajikistan) populations. In conclusion, we consider it is preferable to keep the synonymy.

**Table 2.** Metric and meristic features of *Aphidura ornatella*, and *Aphidura delmasi*; n, number of measured specimens.<br/>

	***Aphidura ornatella***	***Aphidura delmasi***	***Aphidura delmasi***	***Aphidura delmasi***
	**Al. viv. femal.**	**Fundatrices**	**Ovip. femal.**	**Males**
	**n = 6**	**n = 4**	**n = 4**	**n = 4**
Body [mm]	1.637–2.100	1.700–2.200	1.625–1.775	1.175–1.425
Antenna [mm]	1.565–2.060	1.115–1.500	1.660–1.825	1.555–1.725
Antenna / Body [times]	0.96–1.11	0.65–0.68	1.01–1.12	1.20–1.44
Ant. segm. III [mm]	0.40–0.58	0.28	0.44	0.40–0.41
Ant. segm. IV [mm]	0.24–0.36	0.16–0.24	0.29–0.37	0.27–0.33
Ant. segm. V [mm]	0.21–0.29	0.15–0.23	0.25–0.26	0.21–0.25
Ant. segm. VI base [mm]	0.11–0.13	0.11–0.13	0.12–0.13	0.11–0.12
Ant. segm. VI processus terminalis [mm]	0.50–0.67	0.24–0.29	0.44–0.48	0.40–0.50
Ant. segm. VI processus terminalis/ Ant. segm. III [times]	1.09–1.24	0.62–0.93	1.1–1.20	1.00–1.22
Ant. segm. VI processus terminalis/ base [times]	4.62–5.36	1.96–2.48	3.62–4.04	3.48–4.30
Secondary sensoria, Ant. segm. III [number]	21–28	0	0	0
Ultimate rostral segm. [mm]	0.12–0.15	0.13–0.15	0.15–0.17	0.14
Ultimate rostral segm. / its basal width [times]	2.09–3.00	2.00–2.25	2.50–2.82	2.00–2.80
Ultimate rostral segm. / Ant. segm. VI base [times]	1.08–1.16	1.12–1.29	1.15–1.35	1.17–1.33
Hind tarsus, 2nd segm. [mm]	0.11–0.14	0.09–0.10	0.01–0.11	0.08–0.09
Hind tarsus, 2nd segm. / Ultimate rostral segm. [times]	0.93–1.04	0.67–0.70	0.65–0.71	0.57–0.67
Abdominal Marginal papillae [number]	0	0	0	0
Siphunculus [mm]	0.38–0.42	0.23–0.31	0.29–0.32	24–0.26
Siphunculus / Body [times]	0.19–0.23	0.13–0.15	0.17–0.20	0.17–0.21
Siphunculus / Ant. segm. III [times]	0.68–0.95	0.71–0.83	0.72–0.80	0.59–0.66
Siphunculus / its basal width [times]	5.07–6.25	3.22–4.43	4.41–4.75	4.00–4.64
Siphuncular widths, maximal / basal [times]	0.80–1.25	0.72–0.86	0.83–0.92	0.80–0.91
Siphuncular widths, maximal / minimal [times]	1.47–2.33	1	1	1
Cauda [mm]	0.13–0.19	0.15–0.22	0.18–0.19	0.10–0.15
Cauda / Siphunculus [times]	0.34–0.51	0.67–0.74	0.58–0.63	0.37–0.60
Cauda / its basal width [times]	1.05–1.41	1.20–1.43	1.29–1.42	0.68–1.33
Setae on …				
… Frons [μm]	21–28	35–50	45–55	35–45
… Frons / b. d. Ant. segm. III [times]	1.1–1.8	1.6–2.5	2.0–2.8	1.8–2.3
… Ant. segm. III [μm]	12–20	17–23	22–25	17–23
… Ant. segm III / b. d. Ant. segm. III [times]	0.7–1.1	0.9–1.0	1.0–1.3	0.9–1.3
… Ultimate rostral segm. [number]	14–17	5–8	8–12	9–12
… Abdominal segm. 8 [μm]	25–35	35–45	45–50	38–40
… Abdominal segm. 8 / b. d. Ant. segm. III [times]	1.3–2.0	1.6–2.3	2.0–2.4	2.0–2.1
… Abdominal segm. 8 [number]	4–5	4–7	4–5	3–6
… Genital plate, discal [number]	2–4	2–8	7–10	–
… Genital plate, marginal [number]	10–16	12–13	17–21	–
… Cauda [number]	6–8	6–8	8–10	69

NOTE. Used abbreviations: Al., Alate; Ant., Antennal; b. d., basal diameter; femal., females; Ovip., Oviparous; segm., segment; viv., viviparous.

The species pair *Aphidura mingens* and *Aphidura picta* provides a similar situation: they are considered synonymous names by Eastop and Blackman (2005) and valid names by Kadyrbekov (2013), who found differences in the siphunculi shape (slightly swollen in *picta* specimens and subcylindrical, more or less tapering and curved outwards in *mingens* specimens) and in several quantitative characters. The original description of *Aphidura picta* is quite unsatisfactory because it is based on one specimen «*untypical of the species as a whole*» (Eastop and Blackman, op. cit), which «*might be a fundatrix of that species* [*Aphidura ornata*]» (Hille Ris Lambers 1956), and the species is so variable in sclerotisation, pigmentation, siphuncular shape and setal length (Fig. 1C).

In our opinion the synonymy can stand, because V. F. Eastop studied a wide number of specimens from diverse provenances (host plants, localities and dates), including types (R. L. Blackman, pers.com.), and also because of our observations, or at least it should be maintained in the sense that there is only one variable species involved. Nevertheless the valid name for this species could be *Aphidura mingens* if the holotype of *Aphidura picta* could be shown to be a fundatrix of *Aphidura ornata*, in which case *Aphidura picta* would be a synonym of that species.

Blackman and Eastop (2006) showed that *Aphidura mordvilkoi* and *Aphidura prinsepiae* could be synonyms. Kadyrbekov (2013) established the synonymy. We have found differences between specimens identified as *prinsepiae* by Patshchenko and other ones identified as *mordvilkoi* by Shaposhnikov (Fig. 1D), similar to those shown by Kadyrbekov (2013). All of them can be considered to be a consequence of intraspecific variability. In conclusion, the established synonymy can be kept.

### New host plant and country records

Collection data for the following first records are shown in Table 1.

*Aphidura bozhkoae* (Fig. 2A) is recorded for the first time on *Prunus spinosa* and on *Prunus prostrata*; it was previously recorded from several other species habitually placed in *Prunus*, although some of them can be classified in *Cerasus* or in *Aflatunia*.

*Aphidura delmasi* (Fig. 2B) is recorded for the first time on *Silene viscosa*; it has previously been recorded on other species of *Silene*.

*Aphidura ornata* (Fig. 2C) is recorded for the first time on *Silene inaperta*, *Silene nutans* and *Silene saxifraga*; it has been recorded previously on four other species of *Silene*.

*Aphidura pannonica* (Fig. 2D) is recorded for the first time on *Gypsophila paniculata*; this aphid has been previously recorded from several species of *Silene*.

*Aphidura picta* (Fig. 1C) is recorded for the first time (i) on *Silene glauca* and (ii) from Afghanistan. This aphid has been recorded on several species of *Silene*, and also of *Dianthus*; and it was known from Iran, Tajikistan and Pakistan, and other Asiatic and European countries.

### *Aphidura pujoli*, amendment of distribution

The identification made by G. Remaudière, of four apterous viviparous females belonging to his sample 014072 from Pakistan, as *Aphidura pujoli* is not correct; in fact these specimens belong to a new species, *Aphidura pakistanensis*. In consequence the record of *Aphidura pujoli* from Pakistan by Naumann-Etienne and Remaudière (1976) is incorrect, and *Aphidura pujoli* (Fig. 3A) remains restricted to Europe, having been recorded from Portugal, Spain, France (including Corsica), Switzerland, Italy (including Sicily), and Ukraine.

### *Aphidura delmasi*, new morphs

Collecting data in Table 1: fundatrix, sample 7876; oviparous females and males, sample 7591.

**Fundatrix**. From 4 specimens (Fig. 3B). Very similar to the fundatrigenous aptera described by Remaudière and Leclant (1965) and illustrated in detail by Mme. M. Arnault (page 719, figures 1–8), with shorter antennae, legs and siphunculi, as is normal in fundatrices, and without postsiphuncular sclerites. Metric and meristic features in Table 2.

**Oviparous female**. From 4 specimens (Fig. 3C). Very similar to the fundatrigenous aptera, with paler antennae, yellowish legs (only tarsi are smoky). Hind tibiae not swollen, with 20–30 scent plates. Metric and meristic features in Table 2.

**Male**. From 4 specimens (Fig. 3D). Apterous. Also very similar to the fundatrigenous aptera, but smaller, with paler legs (only tarsi are smoky) and longer antennae. Aedeagus and parameres brown. Metric and meristic features in Table 2.

### New species

Six new species are established: *Aphidura gallica* and *Aphidura amphorosiphon*, which live on species of *Silene*, *Aphidura pakistanensis*, *Aphidura graeca* and *Aphidura urmiensis*, which live on other caryophyllaceous plants (respectively species of *Dianthus*, *Gypsophila* and *Spergula*), and *Aphidura iranensis*, which lives on *Prunus*.

#### Aphidura gallica sp. n.

urn:lsid:zoobank.org:act:7C59AAC8-D440-4EAC-B376-047DA69F053A

http://species-id.net/wiki/Aphidura_gallica

##### Apterous viviparous female

(Figs 4A, B). Colour in life unknown. Head yellowish brown with rugosity near the eyes. Antennal segments I-IV as pale as cephalic dorsum, and V and VI darker. Dorsal pigmentation and sclerotisation very variable. In several specimens, holotype included prothorax with complete but pale band, metathorax with brown spinopleural transverse band and setiferous marginal sclerites, abdominal segments 1-6 with an extensive dark spinopleural patch, partially fragmented in midline and with an irregular lateral margin partly incorporating the marginal sclerites; abdominal segments 7 and 8 with bands paler than patch. In other specimens, dorsum mainly membranous, with pale brown to brown pleural sclerites on abdominal segments 1-6, which are irregular in shape and sometimes joined between segments, and several very small and pale marginal setiferous sclerites. Other specimens have an intermediate degree of sclerotisation and pigmentation. Mesosternal mammariform processes yellowish, thin and tall. Siphunculi cylindrical, dark, and densely covered with denticulate scales. Cauda triangular (sometimes with a slight constriction) with pointed apex, and not darker than legs. Anal and genital plates as pale as cauda. Metric and meristic features in Table 3.

**Table 3.** Metric and meristic features of *Aphidura gallica* sp. n. and *Aphidura amphorosiphon* sp. n.; n, number of measured specimens.<br/>

	***Aphidura gallica***	***Aphidura gallica***	***Aphidura amphoro-siphon***	***Aphidura amphoro-siphon***	***Aphidura amphoro-siphon***
	**Apt. viv. femal.**	**Al. viv. femal.**	**Ap. viv. femal.**	**Al. viv. femal.**	**Male**
	**n = 30**	**n = 2**	**n = 12**	**n = 3**	**n = 1**
Body [mm]	1.475–2.325	1.600–1.725	1.275–1.638	1.100–1.525	1.275
Antenna [mm]	1.070–1.735	1.343–1.343	0.978–1.170	1.265–1.615	1.340–1.380
Antenna / Body [times]	0.66–0.93	0.84–0.84	0.71–0.89	1.05–1.15	1.05–1.08
Ant. segm. III [mm]	0.32–0.49	0.23–0.36	0.26–0.35	0.35–0.40	0.38–0.38
Ant. segm. IV [mm]	0.16–0.36	0.12–0.22	0.14–0.20	0.20–0.28	0.22–0.23
Ant. segm. V [mm]	0.14–0.26	0.11–0.18	0.13–0.18	0.16–0.23	0.17–0.19
Ant. segm. VI base [mm]	0.08–0.13	0.10–0.10	0.09–0.11	0.10–0.13	0.10–0.11
Ant. segm. VI processus terminali mm]	0.25–0.44	0.37–0.37	0.25–0.31	0.32–0.48	0.37–0.38
Ant. segm. VI processus terminalis/ Ant. segm. III [times]	0.66–0.99	1.04–1.04	0.77–1.07	0.85–1.22	1.00
Ant. segm. VI processus terminalis/ base [times]	2.88–4.08	3.89–3.89	2.57–3.08	3.37–4.04	3.57–3.70
Secondary sensoria, Ant. segm. III [number]	0	16–34	0	16–21	63–72
Secondary sensoria, Ant. segm. IV [number]	0	0	0	0	29–32
Secondary sensoria, Ant. segm. V [number]	0	0	0	0	11–15
Ultimate rostral segm. [mm]	0.11–0.15	0.10–0.13	0.12–0.14	0.10–0.13	0.10
Ultimate rostral segm. / its basal width [times]	2.00–3.00	2.00–2.60	2.18–2.80	2.88–2.88	2.67
Ultimate rostral segm. / Ant. segm. VI base [times]	1.10–1.87	1.37–1.37	1.12–1.42	0.96–1.05	0.95–1.00
Hind femur [mm]	0.39–0.60	0.39–0.50	0.35–0.46	0.38–0.45	0.42–0.43
Hind tibia [mm]	0.72–1.13	0.81–1.02	0.61–0.83	0.80–1.00	0.82–0.85
Hind tibia / Body [times]	0.43–0.59	0.51–0.59	0.45-0.55	0.58–0.73	0.64–0.67
Hind tarsus, 2nd segm. [mm]	0.10–0.12	0.11–0.11	0.08–0.10	0.10–0.11	0.09–0.10
Hind tarsus, 2nd segm. / Ultimate rostral segm. [times]	0.70–0.95	0.81–1.05	0.67–0.83	0.83–0.95	0.90–0.95
Abdominal marginal papillae [number]	0	0	0	0	0
Siphunculus [mm]	0.35–0.50	0.35–0.42	0.27–0.33	0.25–0.30	0.24
Siphundulus / Body [times]	0.20–0.29	0.22–0.24	0.18–0.24	0.18–0.24	0.19
Siphunculus / Ant. segm. III [times]	0.87–1.24	0.99–1.84	0.90–1.04	0.65–0.77	0.64–0.65
Siphunculus / its basal width [times]	4.00–5.57	7.00–7.00	3.60–5.80	3.85–5.89	5.33
Siphuncular widths, maximal / basal [times]	0.50–0.86	0.80–0.80	0.83–1.20	0.81–1.11	1.33
Siphuncular widths, maximal / minimal [times]	1.00–1.00	1.00–1.00	1.25–1.79	1.47–2.00	2.18
Siphuncular minimal width / Hind tibia, diameter at middle [times]	1.05–1.57	1.33–1.69	1.00–1.57	1.00-0.50	0.61
Cauda [mm]	0.15–0.24	0.15–0.19	0.14–0.19	0.09–0.15	0.09
Cauda / Siphunculus [times]	0.36–0.50	0.41–0.46	0.45–0.62	0.34–0.50	0.38
Cauda / its basal width [times]	1.07–1.32	1.12–1.36	1.10–1.46	0.86–1.45	0.78
Setae on …					
… Frons [μm]	26–45	23–23	17–38	15–21	10
… Frons / b. d. Ant. segm. III [times]	1.2–2.3	1.6–1.6	1.0–1.9	0.8–1.2	0.5
… Vertex [μm]	23–35	18–18	10–23	15–20	13
… Vertex / b. d. Ant. segm. III [times]	1.00–1.75	1.3–1.3	0.6–1.3	0.8–1.1	0.7
… Ant. segm. III [μm]	13–25	15–20	7–13	7–10	5
… Ant. segm III / T. Ant. segm. III [times]	0.6–1.3	0.7–1.5	0.5–0.7	0.4–0.6	0.3
… Ultimate rostral segm. [number]	10–16	11–11	11–17	9–13	12
… Hind femur, dorsal [μm]	13–25	13–20	7–15	10–15	10
… Hind femur, ventral [μm]	23–45	25–28	20–30	17–23	15
… Hind tibia, dorsal, at middle [μm]	25–38	25–28	20–30	20–23	20
… Hind tibia, dorsal / Tibial diameter (at middle) [times]	0.5–1.0	0.8–0.9	0.6–1.0	0.8–0.9	0.6
… Hind tarsus, first segm. [number]	2–3	2–3	2–3	2–3	2–3
… Abdominal segm. 2–4 [μm]	13–23	16–20	7–13	10–13	7
… Abdominal segm. 2–4 / T. Ant. segm. III [times]	0.6–1.1	0.7–1.5	0.4–0.8	0.5–0.6	0.4
… Abdominal segm. 8 [μm]	23–38	23–28	22–33	18–25	23
… Abdominal segm. 8 / T. Ant. segm. III [times]	1.1–2.0	1.2–1.6	1.0–2.0	1.0–1.3	1.1
… Abdominal segm. 8 [number]	4–5	4–4	4–4	4–4	4
… Ventro-abdominal [μm]	25–50	28–33	20–35	25–33	23
… Genital plate, discal [number]	2–4	2–2	2–2	2–2	—
… Genital plate, marginal [number]	10–17	10–12	10–16	8–10	—
… Cauda [number]	6–9	8–8	6–7	6–6	6

NOTE. Used abbreviations: Al., Alate; Ant., Antennal; Apt., Apterous; b. d., basal diameter; femal., females; n, number of measured specimens; segm., segment; viv., viviparous.

##### Alate viviparous female

(Fig. 4C). Head brown, as dark as thorax. Abdominal segments 1 and 2 with spinal sclerites; segments 4-5 with spinopleural patch, sometimes partially joined with the spinopleural bands on 3 and 6; segments 1-6 with marginal sclerites; segment 7 with a band paler than previously mentioned sclerites; segment 8 with pale setiferous sclerites, sometimes coalesced together. Metric and meristic features in Table 3.

##### Types.

Holotype: Apterous viviparous female (specimen 2), on *Silene gallica*, Banyuls-sur-Mer (Pyrénées Orientales), France, 11-VII-1957, Remaudière *leg*. (sample 4241). Paratypes: 39 apterous and 9 alate viviparous females with the same data that the holotype; plus 49 apterous viviparae on *Silene paradoxa*, Défilé de l’Inzecca (Haute Corse), 4-VI-1970, F. Leclant *leg.* (sample 4660) [Remaudière sample 17925].

##### Etymology.

The specific name of the new species, *gallica*, is an adjective that refers to the Galia, France in times of the Roman Empire, in feminine; it is also coincident with the specific name of the host plant of the holotype.

##### Discussion.

See the discussion of the following new species.

#### Aphidura amphorosiphon sp. n.

urn:lsid:zoobank.org:act:812E7A5A-0BE0-4424-8661-38A7D0CC268C

http://species-id.net/wiki/Aphidura_amphorosiphon

##### Apterous viviparous female

(Fig. 4D). Colour in life unknown. Head yellowish brown to brown. Antennal segments II-III or II-V pigmented like cephalic dorsum, and I and IV-VI or only VI darker than others. Mesosternal mammariform processes rounded, low and pale. Several specimens (holotype included) are pale in general with dark brown intersegmental sclerites, brown postsiphuncular and spiracular sclerites, pale brown setiferous sclerites on abdominal segments 6–8, sometimes coalescing together into transverse bands. The most pigmented specimen has a transverse spinopleural band on prothorax, fragmented bands on mesothorax and abdominal segments 1, 6 and 7, fragmented spinopleural patches on abdominal segments 2–5, and setiferous sclerites on metathorax and abdominal segments 1 and 8. Siphunculi markedly swollen, with stem nearly smooth, and pale or with a smoky apical portion. Cauda triangular, sometimes with a slight constriction near the base, and as pale as the greater part of siphunculi and legs. Genital and anal plates as pale as cauda. Metric and meristic features in Table 3.

##### Alate viviparous female

(Fig. 5A). Head brown, as pigmented as pro- and pterothorax and darker than antennae, tarsi and distal portions of femora and tibiae. Abdominal segments 3–5 with a spinopleural patch, and 7–8 with transverse bands. Siphunculi as dark as pigmented parts of femora. Other qualitative features as in apterae. Metric and meristic features in Table 3.

##### Male.

Winged. Qualitatively very similar to alate viviparous females; with dark parameres. Metric and meristic features in Table 3.

##### Types.

Holotype: Apterous viviparous female (specimen 5), on *Silene* sp. Kuh-e Dinar (Kohgiluyed and Boyer-Ahmad), Iran, 14-IX-1955, Remaudière *leg*. (sample i1118a). Paratypes: 15 apterous, 2 viviparous females and 1 male with the same data that the holotype; plus 1 apterous viviparae and 2 alate viviparae on an unidentified species of Caryophyllaceae, Chalus [road to Amol] (Mazenderan), Iran, 3-V-1963, Remaudière *leg.* (sample i2417).

##### Etymology.

The specific name is a neutral noun in apposition, formed for the Greek words “amphora” and “siphon”, which respectively mean flask and siphon, like in the genus *Amphorosiphon*.

##### Discussion.

The distinctive features of *Aphidura amphorosiphon* sp. n. and *Aphidura gallica* sp. n. are summarized in the identification key to apterae of *Aphidura* in the general discussion, and in the following modification to the key to aphids on *Silene* (Blackman and Eastop 2006) for addition of *Aphidura amphorosiphon* and *Aphidura gallica* (*Aphidura* spp. from Iran and from France respectively in that key), and also *Aphidura massagetica* and *Aphidura nomadica*, which have been recently described (Kadyrbekov 2013), with deletion of couplets 28 to 34, although several propositions have been partially or completely reutilised:

**Table:** 

27	Anterior part of mesosternum without mammariform processes [rest of the proposition without modification]	*Volutaphis schusteri*
–	Anterior part of mesosternum with a pair of mammariform processes [rest of the proposition without modification]	35
35	SIPH markedly clavate (distal maximum width habitually at least 1.2 times basal minimum)	36
–	SIPH cylindrical, subcylindrical, tapering from base to apex (sometimes outward curved), or slightly clavate (distal maximum width at most 1.2 times basal minimum width)	38
36	Tergum with an extensive almost solid black shield extending over metanotum and ABD TERG 1–6, usually incorporating marginal sclerites	*Aphidura ornatella*
–	Tergum pale or with variable sclerotisation, sometimes extensive but with large windows spinally and marginally, not forming a solid black shield	37
37	ANT PT/BASE 2.55–3.1. RIV+V 1.2–1.5 times HT II. SIPH light with smoky apex. Cauda 1.1–1.5 times its basal width, with 6–7 hairs. Abdomen variably sclerotised and pigmented	*Aphidura amphorosiphon*
–	ANT PT/BASE 3.2–3,9. RIV+V 1–1.2 times HT II. SIPH uniformly pigmented. Cauda 1.1–1.2 times its basal width, with 7–11 hairs. ABD TERG 1–6 with a dark central patch and marginal sclerites	*Aphidura nomadica*
38	SIPH pale or dusky, slightly clavate, 1.5–1.8 times cauda, which is short triangular. Tergum without sclerotisation, completely pale	*Aphidura pujoli*
–	SIPH brown to black at least in part, sometimes slightly clavate, 1.9–2.8 times cauda. Tergum with variable sclerotisation and pigmentation, rarely complete pale	39
39	ABD TERG 2–3 with longest hairs 35–55 μm long, 1.5–2.0 times ANT BD III. ANT I long, 1.3–1.5 times its maximal width. Dorsal abdomen with a large central oval sclerite on ABD TERG (1)2–5	*Aphidura delmasi*
–	ABD TERG 2–3 with longest hairs 4–25 μm long, 0.2–1.1 times ANT BD III. ANT I short, 1.1 times its maximal width at most. Dorsal abdomen with variable sclerotisation and pigmentation, but rarely with a central oval sclerite on ABD TERG 2–5	40
40	Tergum with an extensive almost solid black shield extending over metanotum and ABD TERG 1–6, usually incorporating marginal sclerites. Cauda dark broad triangular, longer than 2 times its basal width and usually shorter than 0.5 times SIPH, and with 10–16 hairs	*Aphidura ornata*
–	Tergum pale or with variable sclerotisation, sometimes extensive but with large windows spinally and marginally, not forming a solid black shield. Cauda variable in shape, proportions and colour	41
41	Cauda tongue-shaped, 1.4–1.8 times its basal width	42
–	Cauda triangular, although sometimes with a slight constriction, 1.05–1.4 times its basal width	43
42	ANT PT/BASE 4.0–5.7. Hairs on ANT III and ABD TERG 2–3 minute, maximally 4–7μm long, 0.15–0.3 times BD III. SIPH 2.2–2.8 times cauda	*Aphidura pannonica*
–	ANT PT/BASE 2.5–4.0. Hairs on ANT III and ABD TERG 2–3 maximally 8–22μm, 0.4–1.0 times BD III. SIPH 1.9–2.5 times cauda	*Aphidura picta*
43	RIV+V 0.9–1.0 times HT II, with 8–10 accessory hairs. Cauda 1.3–1.4 times its basal width. Hairs on ABD TERG 2–3 8–11 μm long, 0.3–0.5 times BD III	*Aphidura massagetica*
–	RIV+V 1.05–1.45 times HT II, with 10–16 accessory hairs. Cauda 1.05–1.35 times its basal width. Hairs on ABD TERG 2–3 13–23 μm long, 0.6-1 times BD III	*Aphidura gallica*

#### Aphidura pakistanensis sp. n.

urn:lsid:zoobank.org:act:D3B0B038-D4A8-41E8-803A-0D2CFE97A8CE

http://species-id.net/wiki/Aphidura_pakistanensis

##### Apterous viviparous female

(Fig. 5B). Colour in life unknown. Antennae, rostrum, legs, siphunculi, genital plate and cauda yellowish. Frontal tubercles low. Mesosternal mammariform processes low, rough and pale, sometimes inconspicuous. Dorsum of thorax and abdomen without segmental sclerites; intersegmental and spiracular sclerites inconspicuous. The characteristic spinules on postsiphuncular area and tergum of abdominal segments 7 and 8 are dispersed and delicate. Siphunculi short, slightly swollen and densely covered with scales. Cauda short triangular, with broad basis. Metric and meristic features in Table 4.

**Table 4.** Metric and meristic features of apterous viviparous females of *Aphidura pakistanensis* sp. n., *Aphidura graeca* sp. n., *Aphidura urmiensis* sp. n., and *Aphidura iranensis* sp. n.; n, number of measured specimens.<br/>

	***Aphidura pakistanensis***	***Aphidura graeca***	***Aphidura urmiensis***	***Aphidura iranensis***
	**n = 4**	**n = 1**	**n = 20**	**n = 6**
Body [mm]	1.725–1.850	1.838	1.900–2.125	1.100–1.300
Antenna [mm]	1.053–1.655	1.670	1.333–1.755	1.005–1.210
Antenna / Body [times]	0.59–0.89	0.91	0.67–0.84	0.80–1.08
Ant. segm. III [mm]	0.32–0.49	0.37–0.40	0.39–0.52	0.30–4.33
Ant. segm. IV [mm]	0.15–0.26	0.26–0.26	0.21–0.34	0.16–0.20
Ant. segm. V [mm]	0.14–0.20	0.19–0.2	0.15–0.21	0.14–0.16
Ant. segm. VI base [mm]	0.11–0.14	0.14	0.09–0.12	0.08–0.10
Ant. segm. VI [mm]	0.22–0.31	0.56	0.30–0.44	0.31–0.35
Ant. segm. VI processus terminalis/ Ant. segm. III [times]	0.63–0.80	1.51	0.65–0.95	0.96–1.13
Ant. segm. VI processus terminalis/ base [times]	2.00–2.39	4.00	2.78–4.00	3.18–4.53
Ultimate rostral segm. [mm]	0.10–0.12	0.13	0.13–0.15	0.10–0.11
Ultimate rostral segm. / its basal width [times]	1.10–2.00	1.86	2.36–2.64	2.20–2.50
Ultimate rostral segm. / Ant. segm. VI base [times]	0.85–1.00	0.93	1.17–1.61	1.08–1.26
Hind femur [mm]	0.39–0.52	0.55–0.54	0.48–0.59	0.33–0.37
Hind tibia [mm]	0.73–0.95	0.92–0.92	0.86–1.05	0.63–0.70
Hind tibia / Body [times]	0.41–0.51	0.50–0.50	0.44–0.52	0.49–0.62
Hind tarsus, 2nd segm. [mm]	0.11–0.13	0.14–0.13	0.10–0.12	0.09–0.10
Hind tarsus, 2nd segm. / Ultimate rostral segm. [times]	1.05–1.20	1.04–1.00	0.69–0.81	0.84–0.95
Abdominal Marginal papillae [number]	0	0	0	(0)2–4
Siphunculus [mm]	0.17–0.20	0.39	0.47–0.58	0.26–0.31
Siphundulus / Body [times]	0.09–0.11	0.21	0.23–0.29	0.21–0.27
Siphunculus / Ant. segm. III [times]	0.41–0.56	1.04	1.11–1.36	0.79–1.02
Siphunculus / its basal width [times]	2.62–3.18	5.50	4.14–5.50	3.86–6.56
Siphuncular widths, maximal / basal [times]	0.69–0.82	0.79	0.44–0.67	0.61–0.89
Siphuncular widths, maximal / minimal [times]	1.00–1.06	1.38	1.00–1.00	1.06–1.13
Siphuncular minimal width / Hind tibia, diameter at middle [times]	1.42–1.50	0.94	1.24–1.65	1.25–1.89
Cauda [mm]	0.11–0.14	0.23	0.18–0.24	0.11–0.12
Cauda / Siphunculus [times]	0.55–0.82	0.60	0.33–0.45	0.37–0.40
Cauda / its basal width [times]	0.71-0.00	1.80	1.20–1.60	0.92–1.05
Setae on …				
… Frons [μm]	8–10	9	33–45	5–10
… Frons / b. d. Ant. segm. III [times]	0.38–0.44	0.39	1.40–2.12	0.25–0.67
… Vertex [μm]	8–10	8	25–40	8
… Vertex / b. d. Ant. segm. III [times]	0.38–0.57	0.3	1.1–1.9	0.4–0.5
… Ant. segm. III [μm]	8–10	10	14–21	5–8
… Ant. segm III / b. d. Ant. segm. III [times]	0.4–0.6	0.4	0.6–0.9	0.3–0.5
… Ultimate rostral segm. [number]	5–7	13	10–15	8–11
… Hind femur, dorsal [μm]	10–13	5	13–20	3–5
… Hind femur, ventral [μm]	13–18	13	25–40	8–10
… Hind tibia, dorsal, at middle [μm]	20–25	18	25–38	15–23
… Hind tibia, dorsal / Tibial diameter (at middle) [times]	0.7–0.8	0.41	0.6–0.9	0.6–0. 9
… Hind tarsus, first segm. [number]	2–3	2–3	2–3	2–3
… Abdominal segm.s 2-4 [μm]	10–10	3	15–23	4–8
… Abdominal segm.s 2-4 / b. d. Ant. segm. III [times]	0.4–0.6	0.11	0.7–1.1	0.2–0.4
… Abdominal segm. 8 [μm]	20–25	10	23–38	8–15
… Abdominal segm. 8 / b. d. Ant. segm. III [times]	1.0–1.4	0.44	1.0–1.8	0.4–0.9
… Abdominal segm. 8 [number]	4–5	2	3–5	4
… Ventro-abdominal [μm]	20–38	28	30–45	11–16
… Genital plate, discal [number]	2	2	2	2
… Genital plate, marginal [number]	10–14	13	9–18	7–10
… Cauda [number]	6–8	7	9–14	6–6

NOTE. Used abbreviations: Ant., Antennal; b. d., basal diameter; n, number of measured specimens; segm., segment.

##### Types.

Holotype: Apterous viviparous female (specimen 1), on *Dianthus* sp. Kalam (Khyber Pakhtunkhwa), Pakistan, 1800 m, 17-VIII-1991, Naumann-Etienne *leg.* [Remaudière’s sample 014072]. Paratypes: 3 apterous viviparous females with the same data that the holotype.

##### Etymology.

The specific name of the new species is an adjective that refers to Pakistan, in feminine.

##### Discussion.

*Aphidura pakistanensis* sp. n. is the third species of the genus living on *Dianthus*. Its distinctive features are summarized in the identification key to apterae of *Aphidura* in the general discussion, and in the following modification to the key to aphids on *Dianthus* (Blackman and Eastop 2006) for addition of *Aphidura pakistanensis*:

**Table:** 

7	ABD TERG 1 and 7 without MTu. SIPH subcylindrical or slightly swollen). Anterior part of mesosternum with a pair of spinal mammariform processes	7A
–	ABD TERG 1 and 7 with MTu. SIPH tapering from base to flange, with no trace of swelling. Anterior part of mesosternum without a pair of spinal mammariform processes	9
7A	Cauda as long as its basal width or shorter. SIPH not longer than 0.20 mm and 0.6 times ANT III. Mesosternal processes small and pale, sometimes inconspicuous. Abdomen without dorsal pigmentation	*Aphidura pakistanensis*
–	Cauda longer than its basal width. SIPH longer than 0.26 mm and 0.60 times ANT III. Mesosternal processes pale or pigmented, always conspicuous. Abdomen pale or variably pigmented	8
8	[without modification]	*Aphidura picta*
–	[without modification]	*Aphidura pujoli*

#### Aphidura graeca sp. n.

urn:lsid:zoobank.org:act:927C4017-2E8C-417E-BDBD-2A3262557025

http://species-id.net/wiki/Aphidura_graeca

##### Apterous viviparous female

(Fig. 5C). Colour in life unknown. Head pale yellow. Antennal segment I-IV and proximal half of V as pale as cephalic dorsum, distal part of V and VI yellow brown. Dorsum of thorax and abdomen membranous and pale, with yellowish brown spiracular and brown intersegmental sclerites. Mesosternal mammariform processes low, rugose and pale. Siphunculi gently and asymmetrically swollen, rugose and more-or-less pigmented like tibiae. Cauda tongue-shaped with broad apex, pigmented like siphunculi. Anal and genital plates as pale as cauda. Metric and meristic features in Table 4.

##### Types.

Holotype: Apterous viviparous female, on *Gypsophila* sp., Veria [road to Kastania] (Imanthia), Greece, 18-VI-1964, G. Remaudière *leg*. (sample 03026).

##### Etymology.

The specific name of the new species is an adjective that means inhabitant of Greece, in feminine.

##### Discussion.

*Aphidura graeca* sp. n. lives on *Gypsophila*, as does *Aphidura gypsophilae*, and also *Aphidura pannonica*, which has been above recorded on this plant-genus for first time. The distinctive features of *Aphidura graeca* are summarized in the identification key to apterae of *Aphidura* in the general discussion and in the following modification to the key to aphids on *Gypsophila* (Blackman and Eastop 2006), to include *Aphidura graeca* and *Aphidura pannonica*, and also *Aphidura naimanica* and *Aphidura togaica*, which have recently been described (Kadyrbekov 2013):

**Table:** 

3	Anterior part of mesosternum with a pair of mammariform processes, ornamented with spinules	3A
–	Anterior part of mesosternum without a pair of mammariform processes	4
3A	SIPH markedly clavate	3B
–	SIPH not markedely clavate	3C
3B	ANT PT at least 1.40 times ANT III. Abdominal dorsum mostly membranous, and pale. SIPH pale	*Aphidura graeca*
–	ANT PT at most 1.20 times ANT III. Abdominal dorsum with pigmented patches and sclerites. SIPH pigmented	*Aphidura naimanica*
3C	Head, prothorax (with a complete or fragmented transversal band) and SIPH brown. Abdominal spinopleural patch variably developed and pigmented and sometimes fragmented or (often in small specimens) absent	*Aphidura pannonica*
–	Head, prothorax and SIPH (sometimes brownish apicad) pale. Abdomen variable sclerotised and pigmented	3C
3D	ANT PT/BASE 3.4-4.4. R IV+V at least 1.0 times HT II	*Aphidura togaica*
–	ANT PT/BASE 5.0-5.5. R IV+V shorter than HT II	*Aphidura gypsophilae*

#### Aphidura urmiensis sp. n.

urn:lsid:zoobank.org:act:41642023-E1EE-4E82-BD61-931EF3866450

http://species-id.net/wiki/Aphidura_urmiensis

##### Apterous viviparous female

(Fig. 5D). Colour in life unknown. Head yellowish brown to brown. Clypeus bigger than those of the other species of *Aphidura*. Antennae yellowish brown, with brown segment VI, distal 1/3 of V, and articulation between IV and V. Mesosternal mammariform processes well separated from one another, pale and round. Intersegmental sclerites small and dark brown; spiracular sclerites on segment 7 wider and darker than other abdominal spiracular sclerites; abdominal segments 3-6 with pleural and sometimes very small setiferous spinal sclerites, or with spinopleural sclerites; abdominal terga 7 and 8 pale. Siphunculi with narrow base, cylindrical (usually with slight outward curve) or slightly swollen, and as pale as tibiae. Cauda tongue-shaped, pale like genital and anal plate. Metric and meristic features in Table 4.

##### Types.

Holotype: Apterous viviparous female (specimen 5), on *Spergula marina*, Shahi island, Lake Urmia (East Azerbaijan), Iran, 5-VIII-1955, Remaudière *leg*. (sample i962). Paratypes: 42 apterous with the same data that the holotype; plus 6 apterous viviparae on *Spergularia marina*, Charimboulaki, Lake Urmia (West Azerbaijan), Iran, 9-VIII-1955, Remaudière *leg.* (sample i004a).

##### Etymology.

The specific name, *urmiensis* is an adjective that refers to lake Urmia, in feminine, from the name of the Catholic Chaldean Archdiocese of Urmia.

##### Discussion.

The distinctive features of *Aphidura urmiensis* sp. n., which lives on *Spergula marina* are summarized in the identification key to apterae of *Aphidura* in the general discussion and in the following modification to key to aphids on *Spergula* and *Spergularia* (Blackman and Eastop 2006) for addition of *Aphidura urmiensis*:

**Table:** 

0	Anterior part of mesosternum with a pair of spinal mammariform processes	*Aphidura urmiensis*
–	Anterior part of mesosternum without a pair of spinal mammariform processes	1

#### Aphidura iranensis sp. n.

urn:lsid:zoobank.org:act:B6A4D5D2-4826-4C5D-AF94-EFE0DCFF4A2C

http://species-id.net/wiki/Aphidura_iranensis

##### Apterous viviparous female

(Fig. 6A). Colour in life unknown. Head brown. Vertex with spinules disposed in scattered groups. Prothorax and at least some of abdominal segment 2-4 with small marginal tubercles; abdominal segment 8 with 0-2, most frequently 1, small spinal tubercles. Mesosternal mammariform processes rounded and pale. Dorsal pigmentation and sclerotisation very variable. In several specimens (holotype included) prothorax with a complete band, mesothorax with a band with lateral windows, metathorax with two large spinopleural sclerites; abdominal segments 1-5 with several setiferous marginal sclerites, and a spinopleural patch, which has irregular edges and windows and may be coalesced with the metathoracic sclerites; abdominal segment 6 with small intersiphuncular and two postsiphuncular sclerites; segments 7 and 8 with brownish band; intersegmental sclerites are embodied in the above; spiracular sclerites inconspicuous. In less sclerotized and paler specimens the bands and patch are broken. Siphunculi slightly swollen, ornamented with denticulate scales, and paler than cephalic dorsum and dorsal thoracic-abdominal sclerotized areas. Cauda thin triangular, paler than siphunculi. Genital plate pale; anal plate coloured like cauda. Metric and meristic features in Table 4.

##### Types.

Holotype: Apterous viviparous female (specimen 1), on *Prunus* sp., Khoy [30 km North] (West Azerbaijan), Iran, 1700 m, 7-VIII-1955, G. Remaudière *leg.* (sample i982). Paratypes: 5 apterous viviparous females, with the same collecting data as holotype.

##### Etymology.

The specific name of the new species, *iranensis*, is an adjective that refers to Iran, in feminine.

##### Discussion.

*Aphidura iranensis* sp. n. is the second species of the genus living on species of *Prunus*. Its distinctive features are summarized in the identification key to apterae of *Aphidura* in the general discussion, and in the following modification to the key to aphids on *Prunus* (Blackman and Eastop 1994) for addition of *Aphidura iranensis*:

**Table:** 

7	[without modification]	8
–	Head capsule with spiculose (sometimes delicate) or nodulose ornamentation	30
32	Anterior part of mesosternum with a pair of spinal mammariform processes, ornamented with spinules (Fig. 89B)	32B
–	[without modification]	33
32B	A continuous sclerotic and dark shield on (metanotum) ABD TERG 1-6(7), including marginal areas; and dorsum of other thoracic segments with sclerotic dark bands. ABD TERG 1-4 without marginal tubercles, and ABD TERG 8 without spinal tubercles	*Aphidura bozhkoae*
–	A continuous dorsal sclerotic shield absent; dorsum of thoracic segments with sclerotic bands, and ABD TERG 1-5(7) with spinal and pleural sclerites or patches, which may be coalescing. ABD TERG 2-4 frequently with marginal tubercles, and ABD TERG 8 frequently with spinal tubercles	*Aphidura iranensis*

## General discussion

The features that distinguish the apterous viviparous females of the *Aphidura* species which share host plants have been described in the modifications to Blackman and Eastop’s keys to aphids on different plant genera (Blackman and Eastop 1994, 2006) in the particular discussion of each new species.

The previously known and the new species together can be distinguished from each other using the following key to apterous viviparous females of species of *Aphidura*. In brackets are: (1) morphological characters that do not have correspondence in the other proposition of the disjunctive, but which are useful to confirm identification; (2) host plants, and distribution data; and (3) illustration reference. In the distribution of each species the countries are in geographical order from West to East, so that a quick general assessment of the distribution of each species can be made. The key uses data of species recently described from the respective original descriptions (Kadyrbekov 2013); other data are from literature and personal observations. *Aphidura melandrii* is accessible by two routes, because several specimens have slightly swollen siphunculi (maximal swollen width at least 1.2 times minimal stem width) and others have conspicuously swollen siphunculi.

### Key to apterous viviparous females of *Aphidura* species of the world

**Table:** 

1	Siphunculi markedly swollen (maximal swollen width at least 1.2 times minimal stem width)	2
–	Siphunculi of different form (cylindrical, subcylindrical, tapering or slightly swollen, see above “generic characters” section)	9
2	Most of dorsal setae placed on conical tubercles. [Dorsum without segmental pigmented sclerotisation. On *Acanthophyllum* sp.; Iran. Fig. 6B]	*Aphidura acanthophylli*
–	Dorsal setae not placed on tubercles	3
3	Mesosternal processes and cauda pale	4
–	Mesosternal processes and cauda more or less pigmented, light brown to brown	6
4	Siphunculi dark brown, 2.3–2.7 times cauda which has 7–11 setae. Abdominal dorsum with spino-pleural patch, postsiphuncular sclerites pigmented and marginal sclerites. [Ultimate rostral segment 1.0–1.2 times second segment of hind tarsi. Cauda 1.1–1.2 times its basal width. On *Silene suffrutescens* and *Silene* sp.; Kazakhstan. Kadyrbekov (2013): fig. 8]	*Aphidura nomadica*
–	Siphunculi pale, sometimes with smoky apex, 1.6–2.2 times cauda, which has 6–7 setae. If a spino-pleural patch present then ultimate rostral segment is 1.2–1.5 times second segment of hind tarsi	5
5	Antennal segment VI processus terminalis at least 1.4 times antennal segment III and approximately 4 times antennal segment VI base. Longest dorsal setae on abdominal segment 2–4 approximately 3 μm. Cauda tongue-shaped. Dorsum pale with dark intersegmental sclerites. [On *Gypsophila* sp.; Grece. Fig. 5C]	*Aphidura graeca* sp. n.
–	Antennal segment VI processus terminalis at most 1.1 times antennal segment III and at most 3.1 times antennal segment VI base. Longest dorsal setae on abdominal segment 2–4 are 7–13 μm. Cauda triangular, sometimes slight constricted. Dorsum with variable sclerotisation and pigmentation, sometimes mostly pale. [On *Silene* sp., and an unidentified caryophyllaceous species; Iran. Fig. 5D]	*Aphidura amphorosiphon* sp. n.
6	Abdominal (or thoracic-abdominal) discal plate present, sometimes divided in transversal bands	7
–	Abdominal discal plate absent; a broken an irregularly edged spinopleural patch usually present, sometimes with bridges to marginal sclerites	8
7	Mesosternal processes wide and low. Longest dorsal setae on abdominal segment 2–4 are 10–11 μm. Discal plate sometimes divided in transversal bands. Siphunculus 1.6–2.0 times cauda, which has 7–11 setae. [On *Melandrium album*; Kazakhstan. Kadyrbekov (2013): fig. 6]	*Aphidura melandrii*
–	Mesosternal processes more or less narrow and tall. Longest dorsal setae on abdominal segment 2–4 are 10–55 μm. Discal plate always complete. Siphunculus 1.6–2.6 times cauda, which has 5–8 setae. [On *Saponaria* sp., *Silene commutata*, *Silene kuschakewiczii*, *Silene lithophila*, *Silene vulgaris*, *Silene wallichiana*, *Silene wolgensis* and *Silene* sp.; Kazakhstan, Pakistan, Tajikistan, and India. Fig. 1A]	*Aphidura ornatella*
8	Siphunculus 1.7–2.7 times cauda. Longest frontal setae 22–28 μm and 1.0–1.4 times basal diameter of antennal segment III. [On *Gypsophila altissima* and *Gypsophila paniculata*; Kazakhstan. Kadyrbekov (2013): fig. 4]	*Aphidura naimanica*
–	Siphunculus 1.5–1.7 times cauda. Longest frontal setae 35–40 μm and 1.6–1.8 times basal diameter of antennal segment III. [On *Cerastium cerastoides*; Kazakhstan. Kadyrbekov (2013): fig. 5]	*Aphidura alatavica*
9	First segment of tarsi with 4 or less habitually with 3 setae. [Head and prothoracic transversal band as dark as thoracic-abdominal discal plate. Siphunculi cylindrical and straight. On Rosaceae species]	10
–	First segment of tarsi habitually with 3 setae, sometimes with 2; very infrequently with 4	11
10	Antennal segment VI processus terminalis 2.2–2.7 times antennal segment VI base. Ultimate rostral segment with 2–5 accessory setae. Marginal tubercles usually present on abdominal segments 2–4. [On *Prinsepia sinensis*; Russia (Far Est, Primorsky Krai). Fig. 1D]	*Aphidura mordvilkoi*
–	Antennal segment VI processus terminalis 3.8–4.2 times antennal segment VI base. Ultimate rostral segment with 8–10 accessory setae. Abdominal marginal tubercles always absent. [On *Prunus erythrocarpa*, *Prunus fruticosa*, *Prunus incana*, *Prunus spinosa*, *Prunus tianschanica*, *Prunus triloba*, *Prunus ulmifolia*, *Prunus verrucosa* and *Prunus* sp.; Georgia, Kazakhstan, Iran, Uzbekistan, Tajikistan, and Kyrgyzstan. Fig. 2A]	*Aphidura bozhkoae*
11	Siphunculus slightly swollen with a maximal width close to 1.2 times minimal stem width and 1.6–2.0 times cauda, which is 1.5–1.8 times its basal width and has 7–11 setae; both as dark as head dorsum and thoracic and abdominal sclerotisation (a discal plate can be present). Longest dorsal setae on abdominal segment 2–4 are 10–11 μm and approximately 0.5 times basal diameter of antennal segment III. [On *Melandrium album*; Kazakhstan. Kadyrbekov (2013): fig. 6]	*Aphidura melandrii*
–	Characters not in above combination	12
12	Siphunculus at most 1.95 times cauda (which is short triangular), pale or uniformly dusky and slight swollen. Dorsum of head and mesosternal processes pale. Segmental thoracic and abdominal sclerotisation and pigmentation absent	13
–	Siphunculus at least 1.90 times cauda, both diversely shaped and coloured. Dorsum of head and mesosternal processes pale or pigmented. Thoracic and abdominal segmental sclerotisation and pigmentation rare completely absent	14
13	Siphunculus at least 0.26 mm, 0.6–0.95 times antennal segment III, and 1.7–1.95 times cauda, which is longer than its basal width. Mesosternal processes conspicuous. [On *Dianthus carthusianorum*, *Dianthus caryophyllus*, *Dianthus commutatus*, *Dianthus monspessulanus*, *Dianthus rupicola*, *Dianthus* sp. and *Silene borysthenica*, Portugal, Spain, France, Switzerland, Italy and Ukraine. Fig. 3A]	*Aphidura pujoli*
–	Siphunculus shorter than 0.20 mm, 0.41–0.56 times antennal segment III, and 1.7–1.9 times cauda, which is not longer than its basal width. Mesosternal processes sometimes inconspicuous. [On *Dianthus* sp.; Pakistan. Fig. 5B]	*Aphidura pakistanensis* sp. n.
14	Antennal segment I at least 1.25 times its maximal width. Longest dorsal setae on abdominal segments 2–4 are 35–55 μm and 1.5–2.0 times basal diameter of antennal segment III. [Discal plate oval and dark. Siphunculi weakly ornamented, smooth distad. On *Silene italica*, *Silene nutans*¸ perhaps *Silene viscosa*, and *Silene* sp.; France, Italy, Greece. Fig. 2B]	*Aphidura delmasi*
–	Antennal segment I at most 1.1 times its maximal width. Longest dorsal setae on abdominal segments 2–4 at most 25 μm and 1.2 times basal diameter of antennal segment III	15
15	Abdomen usually with spinopleural patch and separate marginal sclerites; if a discal plate is present then it has irregular margins and frequently there are windows in spinal areas of the thoracic, if integrated, and anterior abdominal segments. Dorsal patch or plate smooth and reticulated. Siphunculi dark brown to black, subcylindrical and usually straight, 1.8–2.0 times cauda, which is broad triangular and has 10–16 setae. Ultimate rostral segment with 6–10 accessory setae. [On *Silene inaperta*, *Silene italica*, *Silene nutans*, *Silene saxifraga*, *Silene otites*, *Silene vulgaris*, *Silene wolgensis* and *Silene* sp.; France, Switzerland, Italy, Hungary, Romania, Ukraine and Russia. Fig. 2C]	*Aphidura ornata*
–	Characters not in above combination	16
16	Longest setae on abdominal segments 2–4 (dorsum) and antennal segment III 3–8 μm and 0.15–0.50 times basal diameter of antennal segment III	17
–	Longest setae on abdominal segments 2–4 (dorsum) and antennal segment III 8–25 μm and 0.15–0.50 times basal diameter of antennal segment III; if they are 8 μm long then marginal abdominal tubercles present or ultimate rostral segment shorter than second segment of hind tarsi	18
17	Siphunculi dark brown, head dorsum, mesosternal processes and cauda brown to dark brown. Ultimate rostral segment 1.15–1.25 times second segment of hind tarsi. Cauda 1.4–1.8 times its basal width. [On *Gypsophila paniculata*, *Silene borysthenica*, *Silene moldavica*, *Silene otites*, *Silene wolgensis* and *Silene* sp.; Slovakia, Hungary, Greece, Ukraine, and Moldova. Fig. 2D]	*Aphidura pannonica*
–	Siphunculi (with smoked apex, head dorsum, mesosternal processes and cauda pale. Ultimate rostral segment as long as second segment of hind tarsi. Cauda 1.0–1.1 times its basal width. [On *Gypsophila perfoliata*; Kazakhstan. Kadyrbekov (2013): fig. 1]	*Aphidura togaica*
18	Marginal tubercles on abdominal segments 2–4 and usually at least 1 spinal tubercle on abdominal segment VIII. [Cauda triangular 0.92–1.05 times its basal width. On *Prunus*. Iran. Fig. 6A]	*Aphidura iranensis* sp. n.
–	Marginal and spinal abdominal tubercles absent	19
19	Siphunculi pale, usually as pale as most part of tibiae	20
–	Siphunculi pigmented, usually darker than most part of tibiae	21
20	Antennal segment VI processus terminalis 5.0–5.5 times antennal segment VI base. Cauda triangular or tongue-shaped with slight proximal constriction. Ultimate rostral segment shorter than second segment of hind tarsi. [On *Gypsophila arenaria*, *Gypsophila paniculata*, *Gypsophila perfoliata*, *Gypsophila* sp.; Slovakia, Hungary, Ukraine, Kazakhstan, Russia (Western Siberia). Fig. 6C]	*Aphidura gypsophilae*
–	Antennal segment VI processus terminalis 2.8–4.0 times antennal segment VI base. Cauda tongue-shaped. Ultimate rostral segment 1.23–1.45 times second segment of hind tarsi. [Clypeus swollen both forward and laterally. On *Spergularia marina*; Iran. Fig. 5D]	*Aphidura urmiensis* sp. n.
21	Cauda tongue-shaped, 1.40–1.80 times its basal width. Mesosternal processes more or less pigmented, usually darker than tibiae. [Thoracic and abdominal sclerotisation variable, usually a spinopleural abdominal patch with irregular edges and windows in several segments, including the posterior ones. Siphunculi pigmented, but usually pale than abdominal sclerotised dorsum. On *Dianthus barbatus*, *Dianthus caryophyllus*, *Dianthus crinitus*, *Dianthus* sp., *Silene conoidea*, *Silene fruticosa*, *Silene italica*, *Silene otites*, *Silene paradoxa*, *Silene thymifolia*, *Silene vulgaris*, and *Silene* sp. Spain, France, Italy, Slovenia, Hungary, Greece, Bulgaria, Turkey, Israel, Iran, Afghanistan, Pakistan, Tajikistan, and Russia (Asiatic part). Fig. 1C]	*Aphidura picta*
–	Cauda triangular, although sometimes with a slight proximal constriction, 1.05–1.40 times its basal width. Siphunculi and mesosternal processes as pale as tibiae	22
22	Ultimate rostral segment 0.90–1.00 times second segment of hind tarsus, with 8–10 accessory setae. Cauda approximately 1.30–1.40 times its basal width. Longest dorsal setae on abdominal segment 2–3 are 8–11 μm and 0.3–0.5 basal diameter of antennal segment III. [On *Silene lithophila*; Kazakhstan. Kadyrbekov (2013): fig. 2]	*Aphidura massagetica*
–	Ultimate rostral segment 1.05–1.45 times second segment of hind tarsus, with 10–16 accessory setae. Cauda approximately 1.05–1.35 times its basal width. Longest dorsal setae on abdominal segment 2–3 are 13–23 μm and 0.6–1.0 basal diameter of antennal segment III. [On *Silene gallica* and *Silene paradoxa*; France. Figs 4A, B]	*Aphidura gallica* sp. n.

*Aphidura pujoli* (from Blackman and Eastop op. cit.) and *Aphidura delmasi* (this paper) are monoecious holocyclic, and *Aphidura amphorosiphon* is very possibly holocyclic (this paper). The life cycle of the other species of the genus is unknown. It is possible that three types of life cycle currently exist in this genus, as in *Brachycaudus* van der Goot, 1913: (i) monoecious (and probably holocyclic) on a rosaceous species (e.g. *Aphidura bozhkoae* on *Prunus* spp. and *Aphidura mordvilkoi* on *Prinsepia sinensis*), (ii) monoecious on a caryophyllaceous species (and also probably holocyclic, e.g. *Aphidura delmasi* and *Aphidura pujoli*), and (iii) dioecious cycle with rosaceous species as primary host, and caryophyllaceous species as secondary host.

For us the more probable hypothesis is that all current species of *Aphidura* are monoecious, but that their common ancestor was dioecious, as in various other genera of Macrosiphini, and later the *Aphidura* branch diversified into two monoecious lineages, one Rosaceae-feeding and the other Caryophyllaceae-feeding. This is analogous to the South American species of *Pentamyzus* Hille Ris Lambers, 1966 which are all monoecious holocyclic, with several species living on *Acaena* (Rosaceae) and others on *Alopecurus*, *Hordeum* or *Poa* (Poaceae) (Nieto Nafría et al. 2002).

## Supplementary Material

XML Treatment for Aphidura gallica

XML Treatment for Aphidura amphorosiphon

XML Treatment for Aphidura pakistanensis

XML Treatment for Aphidura graeca

XML Treatment for Aphidura urmiensis

XML Treatment for Aphidura iranensis
